# How French physicians manage with a future change in the primary vaccination of infants against diphtheria, tetanus, pertussis and poliomyelitis? A qualitative study with focus groups

**DOI:** 10.1186/1471-2296-14-85

**Published:** 2013-06-19

**Authors:** Karine Lungarde, Fanette Blaizeau, Isabelle Auger-Aubin, Daniel Floret, Serge Gilberg, Christine Jestin, Thomas Hanslik, Corinne Le Goaster, Daniel Lévy-Bruhl, Thierry Blanchon, Louise Rossignol

**Affiliations:** 1 INSERM U707, 27, rue Chaligny, Paris F-75012, France; 2 Département de médecine générale, UPMC Université Paris 06, 27, rue Chaligny, Paris cedex 12 75571, France; 3 UPMC Univ Paris 06, UMR S 707, Paris F-75012, France; 4 Sorbonne Paris Cité, Département de Médecine Générale, Université Paris Diderot, Paris F-75018, France; 5 Université Claude Bernard, Lyon 1, Domaine Rockefeller, avenue Rockefeller, Lyon 69008, France; 6 Haut Conseil de la santé publique, Secrétariat général, 11, place des cinq martyrs du lycée Buffon, Paris 75014, France; 7 Faculté de médecine, Département de médecine générale, Université Paris Descartes, 24 rue du faubourg Saint - Jacques, Paris 75014, France; 8 INPES Institut national de la prévention et d’éducation pour la santé, 42 bd de la Libération, Saint-Denis cedex 93203, France; 9 UFR de médecine Paris-Île-de-France-Ouest, université Versailles Saint-Quentin-en-Yvelines, 9, boulevard d’Alembert, Guyancourt 78280, France; 10 Unité des maladies à prévention vaccinale, département des maladies infectieuses, Institut de Veille Sanitaire, (InVS), 12, rue du Val-d’Osne, Saint-Maurice 94415, France

**Keywords:** Qualitative research, Vaccination, Primary care

## Abstract

**Background:**

As in other European countries, the French vaccination schedule changes according to epidemiological and socio-economic situations. Further changes are planned for 2013, including the withdrawal of one dose for primary vaccination against diphtheria, tetanus, polio, pertussis and Haemophilus influenzae. A partnership between the French Technical Vaccination Committee and the French Institute for Health and Medical Research designed a study to assess primary care physicians’ agreement about this modification.

**Methods:**

Qualitative study with focus groups and semi-structured interviews in France. Four focus groups were conducted with physicians, supplemented by four individual interviews.

**Results:**

The physicians of the survey had accepted the suggested vaccination schedule well. A few concerns had been underlined: fear of less follow-up care for infants resulting from the removal of one visit driven by the primary vaccination; fear of loss of vaccine efficacy; suspicion of the existence of financial arguments at the origin of this change; and adjustment to current vaccination schedule. Several suggestions were made: providing strong support from health authorities; developing stable and simple recommendations; providing effective tools for monitoring patient’s vaccination status.

**Conclusions:**

Physicians’ opinions suggested a good acceptance of a possible change about primary vaccination against diphtheria, tetanus, polio, pertussis and Haemophilus influenzae. Physicians’ suggestions resulted from this qualitative study on a new vaccination schedule. It showed how that their involvement was feasible for preparing the implementation of a new vaccination schedule.

## Background

In France, children receive seven doses of vaccine against diphtheria, tetanus and inactive polio virus (DT-IPV) before the age of 18 years, including four doses before the age of 2 years [1]. France is one of the European countries in which children receive the greatest number of injections [2]. Before the age of 2-years old, two vaccination schedules for DT-IPV exist in the world. They recommend either three or four doses. Several studies have suggested that the primary vaccination with three doses for DT-IPV (at the age of 3, 5 and 11–12 months) would be sufficient and effective [3-5]. In 2009, four European countries had adopted this vaccination schedule (Denmark, Norway, Italy and Sweden) [2]. In 2012, the French Technical Vaccination Committee (CTV) had studied the possibility of reducing one dose of DT-IPV vaccine in childhood vaccination before the age of 2 years. This discussion was part of a broader debate including the DT-IPV vaccination after the age of 2 years, but also others vaccinations such as vaccinations against whooping cough or measles This new calendar has been edited in April 2013 and available on the French Institute for Public Health Surveillance website [http://www.invs.sante.fr] [6]. In France, DT-IPV vaccination is compulsory since 1965. Changing this vaccination may represent a deep change. The Public Health High Council and particularly Technical Vaccination Committee are in charge of vaccination strategies development in France. The Ministry of Health validates and implements their notices. Each year a new calendar is edited with past and new recommendations. Even if DT-IPV vaccination’s coverage was 91.5% in 2007, this deep change in DT-IPV vaccination is like a small revolution in France [7]. Because of this, it appears necessary for the CTV to assess primary care physicians’ agreement prior to this modification. This agreement is necessary to get high vaccination coverage [8]. Vaccination against A (H1N1) influenza virus during the 2009 pandemic is a perfect example of the major role of GPs in a mass vaccination [9]: while GPs were initially very supportive of this vaccination [10], public authorities did not involve them in the vaccination campaign for logistical reasons. Patients were vaccinated in hospitals or in dedicated vaccination units. Overall, the vaccination campaign resulted in poor vaccination coverage (7.9%) [11]. In the field of childhood primary vaccination, implementation of measles vaccination confirmed that the new recommendation is a long learning. Since first dose of measles vaccine recommendation in 1983, the vaccine coverage rate of 95% has not been reached [12], and took nearly 10 years to reach 80% (32% in 1985, 80% in 1994) [13].

The first aim of this study was to examine the perceptions of primary care physicians about a change DT-IPV vaccination. Secondary objectives were to understand their needs for the implementation of the new vaccination schedule and to investigate the perceptions of primary care physicians in relation to parental acceptance of the annual changes in vaccination schedule.

## Methods

### Participant recruitment procedure

Qualitative method was used with focus groups and semi-structured interviews [14-16]. These methods are more appropriate than quantitative ones to study the variety of opinions and feelings of the actors, and to generate new hypotheses. The focus groups are a dynamic group of discussions to gather information, assess needs, behaviours, and various views of a target population on a given subject. The semi-structured individual interviews were used to collect similar information from individuals who were not able to participate in focus groups (for example working in rural areas or being too busy). Those methods are used to examine physicians’ immunization behaviour [17-19]. Four focus groups with primary care physicians, supplemented by four semi-structured interviews were performed in France. Two medical specialties take care of children in French primary care: general practitioners (GPs) and paediatricians. In 2011, they respectively represented 96% and 4% of those physicians [20]. It was decided to set up focus groups for these two different specialties.

Focus groups were conducted between April and July 2012, and individual interviews between July and September 2012. The study population was drawn from different regions of France. Participants were selected among local contacts and among physicians belonging to a network (the French GPs Sentinelles network [21], or the French Association of Ambulatory Paediatrics (AFPA)). Physicians who gave their consent were contacted by telephone to answer questions: number of years since installation, proportion of children seen in consultation, practicing complementary and alternative medicine, member of a network and teaching activity. Considering these answers, groups of participants as heterogeneous as possible were built to obtain maximum variation sampling. Each focus group included younger and older, more experienced physicians, male and female, from both urban and rural areas. The focus groups had to gather six to twelve people, recruitment stopped when the number of participants was in this range. Anti-vaccination physicians were excluded from this selection. As they did not participate to vaccination campaign, they would not have been able to bring any interesting information to this study. Vaccination schedule modification would have little or no consequences on a non-vaccinating physician’s practice.

To test data saturation of focus groups (a situation in which data has been heard before), individual semi-structured interviews were conducted with GPs who could not participate in focus groups, which completed the focus groups’ results.

### Data collection

A topic guide was developed (Table 1), checked and validated by the study scientific committee, and tested by telephone with two GPs. This document included fifteen open-ended questions used by the moderators as a frame to animate exchanges. Recovery questions were planned if the moderator noted a decline in the group’s dynamism. The first part of the interview focused on physicians’ opinions and experiences concerning vaccination: their perceptions and practices concerning the current vaccination schedule, as well as motivations and barriers encountered in its implementation. Secondly, the interview gathered physicians’ reactions concerning the modifications planned by the CTV and their wishes for the implementation of this new vaccination schedule. For each focus group, a trained moderator led the discussion to ensure that all topics of the study were discussed and that all physicians were involved in the discussion. Focus groups were observed by a researcher who gathered information on non-verbal communication and interaction between participants. One investigator led all individual interviews by telephone with the same topic guide to avoid methodological bias. Audio recordings of the focus groups and the individual interviews were done, and these recordings were transcribed.

**Table 1:** Topic guide

**Questions**	**Reopening**
**First ice breaking question: we are going to talk about regular changes of the vaccination schedule. At first, could you tell us how do you use the vaccination schedule in your daily practice?**	**Regarding the children/infants? How do you explain it to the parents**
Why do you face some difficulties?	How do you resolve any possible conflicts or misunderstandings?
Do these changes seem justified to you?	How would you explain these changes? Why would it be a problem?
*In order to make the current vaccination schedule easier, a work group of the French Technical Vaccination Committee would possibly* reduce the number of injections administered to infants from 4 to 3 injections between 0 and 18 months of DT-IPV-Ca-Hib^¥^. *This would imply an important modification of the actual vaccination schedule.*	
How, in terms of acceptability, usefulness, feasibility, and impact on practices, would you react to such a change?	Would you need more scientific evidence?
Do you think that this change will be understood or accepted by the family of the children involved (a priori and/or based on past experience)?	What level of proof do you need a priori?
How do you plan to apply these changes?	Would you be willing to incorporate this change into your practice? What would be your condition(s) to apply? If you are not ready or disagree, why?
What are your wishes for the implementation of the revised schedule?	If such a change took place, what are the best ways for you to set it up?
What do you think of the current means to broadcast new recommendations?	What tools would you like to get in order to make access to information easy about the foreseen change?
	What tools are currently available to you? Which ones do you use?
Compared to those you already have, do you think they should be improved? Multiplied?	

^¥^Vaccine against diphtheria, tetanus, inactive polio virus, whooping cough and Haemophilus b.

### Analysis

A thematic approach was used. From the transcript of records, two researchers each created a code list based on the verbatim quotes. These codes were shared and discussed within the research team. The codebook was continuously revised in order to compare all of the codes and to clarify their meaning, going back to the context until mutual consent was reached. The final thematic list was submitted to the Scientific Committee and discussed with its members.

### Ethics statement

This study has been conducted by the Sentinel network. The Sentinel network is regulated by the mixed research unit UMR-S 707 of INSERM and University of Paris VI: Pierre and Marie Curie, in collaboration with the French Institute for Public Health Surveillance (Institut de veille sanitaire, InVS). It has obtained a research authorisation from the French independent administrative authority protecting privacy and personal data (CNIL), n°471 393.The ethical approval from the ‘National Commission for Computing and Liberties’ (‘Opinion No. 471393, September 1996’) had been given for continuous surveillance of health indicators and for some specific studies, which do not involve human participants. Specific ethical approval is not required for this study

## Results

Four focus groups and four individual interviews, involving 45 physicians, were carried out. Data saturation was reached after four focus groups, and the four individual interviews did not bring any additional themes or sub-themes. The selection process resulted in a heterogeneous study population. The characteristics of the participants are summarised in Table 2. Each quote is the comments expressed by one physician, although they can be agreed, moderated or disproved by the other participants. Each quote was chosen because it best represents recurring ideas arising from discussions.

**Table 2:** Physicians’ profiles and practices

	**Primary care physicians**
	**(N = 45)**
Medical specialties: General practitioner / Paediatric	36 / 9
Sex: Male / Female	23 / 22
Age, mean (min-max)	50 (32–66)
Number of years since installation, mean (min-max)	18 (1–35)
Proportion of children of their activity:	
Children under 16-years old (%), mean (min-max)	43 (10-100%)
Working area, n (%)	
Rural practice	14 (31%)
Urban practice	23 (51%)
Mixed practice	8 (18%)
Practicing complementary and alternative medicine n (%)	3(7%)
Group practice n (%)	24 (53%)
Teaching activity n (%)	14 (31%)

### Current vaccination schedule

According to the study’s physicians (Table 3), most patients accepted vaccinations without difficulty (Quote 1). Protection against serious diseases was one of the main reasons to get vaccinated. Some vaccines names can be very persuasive for being vaccinated (Quote 2). A trustworthy relationship between physicians and patients would be helpful (Quote 3). It has been found that a wide dissemination of information on vaccines was useful, particularly through the mass media campaign (Quote 4). Compulsory vaccination appears to promote physicians’ and patients’ compliance with the vaccination schedule. The reimbursement of the vaccines seems to improve the acceptance of vaccination (Quote 5).

**Table 3:** Conditions leading to the easy application of the vaccination schedule

**Quote number**	**Theme**	**Verbatim**
Quote 1	Acceptance of the patient	« I rarely meet people who refuse » *(FGR male, GP, 57, rural practice)*
Quote 2	Vaccines and their power of persuasion	« It’s easier for parents to accept vaccines that are unknown to the public, for instance, the meningococcal C vaccine. Because there is the magical word. The word “meningitis” is scary…! » (FG paediatrician, female, 62, urban practice)
Quote 3	Physician-patient relationship	« They trust their GP, their family physician. If the physician is not that sure, it is less probable that the vaccination will be done » *(FGR, man, GP, 50, mixed practice)*
Quote 4	Broadcasting information	« Meningitis, (…) it is widely understood (…) because there have been cases and this information has been broadcasted » *(FGR, female, GP, 40, rural practice)*
Quote 5	Vaccine reimbursement	« (…) As long as vaccine is reimbursed, [it is easier] to obtain their consent for vaccinations » *(FGR, man, GP, 55, urban practice)*

*GP* General Practitioner, *FG* Focus Group.

Most physicians of this survey encountered difficulties in adapting quickly to regular vaccination schedule changes (Table 4). They noticed that these changes influence patient’s acceptance and induce problems concerning catch-up schedules (Quote 6). Introduction of new vaccines into the vaccination schedule would create more difficulties with respect to the existing vaccine. Finally they seemed to consider that the vaccination schedule got more and more complicated (Quote 7). The participants suggested differences in physicians’ practices. This could cause additional problems in convincing patients of the vaccination’s value (Quote 8). Physicians expressed a lack of scientific evidence for the changes in the vaccination schedule (Quote 9). They wished for a better visibility of this scientific evidence by the Health Authorities. According to them, one recurrent problem was missing opportunities of vaccination. Written materials, such as health records or vaccination cards were often lost or non-existent (Quote 10). Physicians wanted to improve the dissemination of information to patients in order to decrease patient’s refusal and improve awareness of recommendations (Quote 11). The negative effects of the media had probably increased the fear of side effects (Quote 12). According to participants, patients would distrust health authorities (Quote 13). The physicians felt a disengagement of these authorities and would like more support from them.

**Table 4:** Perceived barriers to vaccination

**Quote number**	**Theme**	**Verbatim**
Quote 6	Patient misunderstanding	« At school, people say : “how come you haven’t been vaccinated by your physician ?” (then we answer) “But at that time, it (the vaccination schedule) was different.” To people’s eyes, we’ve become less trustworthy. » (FGF, female, GP, 47, rural practice)
Quote 7	Complexity of indications	« I mean, given the actual criteria encouraging the practice of BCG vaccination (…), telling the patient whether or not to get vaccinated, according to his geographic and ethnic origins, it is not always obvious.» *(FGR, man, GP, 50, mixed practice)*
Quote 8	Different medical practices	« It is difficult for me to give a different medical advice by saying "Hepatitis B vaccine, let’s go for it!" while another physician told the same patient not to get vaccinated immediately » *(FGF, man, GP, 43, mixed practice)*
Quote 9	Lack of scientific justification	« There still isn’t any evidence showing the efficacy of HPV vaccine» *(FGP, man, GP, 57, urban practice)*
Quote 10	Inadequate aftercare	« I met two young girls who (…) came to see me without their child health notebooks. They were 7- and 9-years old, I was wondering which vaccine to administrate? No one could tell me which vaccines they have had since birth. » *(FGF, female, GP, 58, rural practice)*
Quote 11	Insufficient information broadcasting	« I also have the feeling that there is little information broadcasted at the national level, I would say generally that we are the only ones who give this information, thereby losing our credibility as physicians » *(FGR, man, GP, 57, rural practice)*
Quote 12	Negative impact of mass media campaign	« I got the feeling that patients lose trust in medication, especially vaccines. I think that the mass media campaign around the influenza A(H1N1) vaccination led to bad publicity. I experience this unwavering mistrust in my daily practice; for instance, when you were referring to the hepatitis B vaccine episode, which has deeply marked patients and physicians. » *(FGR, man, GP, 50, mixed practice)*
Quote 13	Mistrust of institutions	« As for me, I still have the feeling that there is a great loss of trust from the patients toward the public authorities, be it the media or even the official authorities. » *(FGR, man, GP, 39, rural practice)*

*GP* General Practitioner, *FG* Focus Group.

### The vaccination schedule proposed

Supposing that the number of injections of DT-IPV before the age of 2 years was reduced, all participating physicians had a favourable opinion. According to them (Table 5), children were more anxious and sensitive regarding injections before the age of 2 years. Reducing the number of vaccines for infants would be less problematic than adding an extra dose of vaccine or a new vaccine (Quote 14). The physicians also paid attention to this modification, improving the physician-patient relationship, if vaccine efficacy remains the same. They could see the children and their parents more peacefully when no vaccination was planned. According to them, parents would accept that one dose of vaccine is removed from the vaccination schedule (Quote 15). Vaccinating infants against DT-IPV in a shorter period than the current schedule would be seen as an advantage. The interviewed physicians reported that receiving prime vaccines during the first 12 months would facilitate adhesion to vaccination schedule (Quote 16). A fixed age concerning DT-IPV booster dose for adults was considered as an advantage to facilitate the implementation of the new vaccination schedule (Quote 17). Some physicians reported that they sometimes adapted their vaccination practices before the schedule’s changes, according to scientific reading or personal positions (Quote 18).

**Table 5:** Strengths of the proposed vaccination schedule mentioned by physicians

**Quote’s number**	**Theme**	**Verbatim**
Quote 14	Lowering the frequency of DT-IPV injections	« The interesting thing is that there is no need to repeat the injection at 3 months. This allows me to finally (…)meet them again in 3 months’ time without any vaccination …» *(FGR, female, GP, 40, rural practice)*
Quote 15	Patient adherence	« I think that they [parents] should be happy that there are fewer [injections]» *(FGF, female, GP, 58, rural practice)*
Quote 16	Primary vaccination shorter	« This is an additional motivation for applying such a scheme. » *(FGF, female, GP, 47, rural practice)*
Quote 17	Vaccination at a given age	« Vaccinated at a fixed age, it will be easier than managing the immunization schedule based on time or latency » *(FGR, man, GP, 57, rural practice)*
Quote 18	Anticipatipating some changes	« About the measles-mumps-rubella vaccination : "Well, to be honest, I have anticipated the new recommendations (laughs) I do it for everyone at age nine ! (…) And then it allows me to give the Prevenar ® along with the meningococcal C at 12. So in the calendar you describe here, well, the potentially calendar-to-be, it is the same as my current practice. » *(EI1, man,GP, 32 , rural practice)*

*GP* General Practitioner, *FG* Focus Group.

Physicians questioned the possible risk of less frequent monitoring of children (Table 6). Vaccination was an opportunity for children’s routine physical check-up (Quote 19). For some modifications, the question of vaccine efficacy remained (Quote 20). The physicians paid a lot attention to be sure that vaccination schedule changes would not come from financial influences (Quote 21). Physicians had always difficult to keep up with the frequent changes in vaccination schedule (Quote 22).

**Table 6:** Possible barriers for applying the proposed vaccination schedule

**Quote’s number**	**Theme**	**Verbatim**
Quote 19	Less aftercare for young children	« Would parents bring us their 16/18-months old child if there is no vaccine to do? » (FG paediatrician, female, 58, urban practice)
Quote 20	Doubts about vaccination effectiveness	« It makes me sick to see them vaccinated at nine months, I think that's a false security because they are not protected » (FG paediatrician, female, 62, rural practice)
Quote 21	Fearing a financial motivation	« If it's only tied to an economic reason, then it is not reliable anymore » (FGR, man, GP, 57, rural practice)
Quote 22	Hard catch-up vaccination schedule	« It is difficult to get used to changes » (FGF, female, GP, 57, rural practice)

*GP* General Practitioner, *FG* Focus Group.

All participating physicians wanted to have scientific evidence supporting the vaccine efficacy despite changes to the vaccination schedule and no side effects induced by the latter (Quote 23) (Table 7). In order to implement more easily the new vaccination schedule, physicians wished to have support from the political and health authorities. Physicians wished to enhance communication with patients and health authorities about the importance of vaccines. They frequently mentioned that there was a need to be supported from the health authorities. Some said that their mission was not to convince patients but that it was the health authorities’ responsibility. Mass campaign media for vaccinations could help to reduce anti-vaccine activity (Quote 24). According to some physicians, improving communication around vaccination could facilitate uptake of the vaccination schedule (Quote 25). If patients trust in vaccines and vaccination, physicians would not have difficulties in practicing it. They also suggested that informing health care practitioners about the new vaccination schedule by individual letters would facilitate its implementation (Quote 26). They considered simplifying vaccination policies as a priority, even if it means losing accuracy (Quote 27). They expressed difficulties to read the entire recommendations, which were too longer or too detailed. Improving vaccination educational materials for healthcare providers could more easily identify people who should be vaccinated. Two types of tools were discussed: those for health professionals and those for patient education (Quote 28). They also put forward the efficacy of using computer tools (Quote 29).

**Table 7:** Physicians’ wishes for implementing the proposed vaccination schedule

**Quote’s number**	**Theme**	**Verbatim**
Quote 23	Strong scientific justification	« (…) There are articles every day about the side effects and some people are getting convinced by physicians who are against vaccines. So if we have something to present we need to be unassailable. » (FG, paediatrician, man, 57, urban practice)
Quote 24	Strong support from health authorities	« I think we should take the opportunity to make a good communication campaign, we often hear from the anti-vaccine lobby, it would be good to hear the voice of competent authorities … » *(FG, paediatrician, female, 62, urban practice)*
Quote 25	Informing the population	« Perhaps there should be a communication like has been done about antibiotics … which went well by the way - inform people » (FGF, female, GP, 47, rural practice)
Quote 26	Informing healthcare professionals	« What would be interesting is that as soon as they change something, they broadcast that to all physicians with leaflets and emails we do receive » *(FGF, female, GP, 45, urban practice)*
Quote 27	Simplifying vaccination policies	« It is better to have a simple calendar, easy to apply, that we will apply, rather than an ideal but complicated one, that we will not apply because it is too difficult to do …» *(FGF, man, GP, 55, mixed practice)*
Quote 28	Improving vaccination educational materials	« Those [recommendations] to be sent to patients, they must be simple. So that they get it right, and that it may be a schedule to give to them, and we'd put on the vaccination page, on top of that already exists. " (EI4 man, GP, 65, mixed practice)
Quote 29		« I expect a lot of universal electronic records in this case. Because it may allow us to have eyes on the reality of vaccinations » *(FGR, man, GP, 57, rural practice*)

*GP* General Practitioner, *FG* Focus Group.

## Discussion

French modification of the infants DT-IPV primary vaccination seems possible and acceptable. The preliminary evaluation of the acceptability of this modification by primary care physicians has highlighted some main points that will facilitate the implementation of the new vaccination schedule:

~Scientific justification and health authorities’ support

~Simplicity and stability of vaccine recommendations

~Tools to help management of vaccinations

Physicians needed strong scientific evidences to justify the new vaccination recommendations. These justifications involve health authorities’ support. At the international level, the objectives are based on transparency and clarity on vaccination strategies [22]. Reviews and reports of the Public Health High Council, in charge of vaccination strategies development in France, are available on their website [http://www.hcsp.fr]. Their availability and reading can respect this principle. Even if scientific justification is a determining factor, it is not sufficient in itself, as shown by the example of hepatitis B vaccination. Despite Public Health High Council in 2004 showed no evidence of a link between hepatitis B vaccination and demyelinating diseases, the infants’ vaccination coverage at the age of 24 months reached only 41.9% in 2007 [7].

In 1996, Pathman *et al.* developed a four-step model necessary for the use of clinical guideline recommendations, particularly on paediatric vaccine usage [23]. These four steps are awareness, agreement, adoption and adherence. Adherence was defined for a physician as 90% or greater of their patients received the vaccine as recommended. Mickan *et al.* realised in 2011 a meta-analysis on the implementation of the recommendations in the United States on various medical fields [24]. The authors showed progressive drop off with the proportion dropping off at each step at about 15%. Awareness of the recommendation is only one of these four steps to improve professional’s practice [23]. When changing the DT-IPV infant primary vaccination, health authorities would have to work on each step to obtain high coverage.

The acceptance of this modification was balanced by physician’s needs to have a stable and simple vaccination schedule. Even if regular changes are required to follow the evolution of advanced vaccinology and epidemiological characteristics, their impact on the health professional’s practices should be taken into account. In Great Britain, two years after the cessation of routine BCG vaccination in 2005 and the implementation of targeted vaccination, two-thirds of parents and professionals interviewed were not aware of the new recommendation [25]. About multiplying specific indications, a French study of 2009 on the determinants of BCG vaccination showed that the probability of an eligible child being properly vaccinated increased with the number of instructions known by the physician [26].

From a practical point of view, demand for developing specific tools to help track the vaccination status of their patients was high among the physicians interviewed. A study in the US showed that the use of a computerised medical record increased opportunities for updating children’s vaccinations and vaccinated them earlier [27]. However, the role of computerised vaccination alerts would be uncertain. Two studies in the US have shown a significant increase in vaccination coverage through the use of computerised vaccination alerts in obstetrics and gynaecology, and rheumatology departments [28,29]. In primary care, this effect has not been demonstrated in a study [30]. It would be interesting to evaluate the effectiveness of computer tools in the modification of the DT-IPV infant’s primary vaccination. With the development of innovative tool like mesvaccins.net, vaccination coverage may be better. This application can be used in France both by physicians and patients to know when and which vaccine are recommended. It is update regularly. For nowadays it is not available for medicine software. This could be something to work.

Asking upstream the local providers about vaccination schedule changes is innovative. Another strength of the study was to obtain heterogeneous focus groups, supplemented by four semi-structured interviews. This permitted to raise most important barriers to implementation of the new calendar. Otherwise the study has several limitations. First, the sampling method might have caused selection bias, including physicians belonging to a network (the French GPs *Sentinelles* network, or the French Association of Ambulatory Paediatrics (AFPA)). Second, physician has indirectly described parents’ demands, in addition of their own perception. Other study should be done to confirm these results. Third, the qualitative data might have been influenced by interpretation bias, despite efforts to reduce such bias. These included double data analysis and discussion of the data with the research team. Fourth, the results of the collection of observational data (non-verbal behaviour of the participants) have not been fully exploited.

## Conclusion

This qualitative study provides an overview of physicians ‘perceptions about a change in the DT-IPV infants’ primary vaccination. The most important result was the favourable opinions of the physicians towards this change. To implement this change, they suggested some interventions, as communication campaign to both physicians and parents, and strong scientific background. This positive experience has enabled collaboration between practitioners and those responsible for developing recommendations.

## Competing interests

Drs Floret, Gilberg and Hanslik are members of the CTV. Dr Floret is the chairman of this committee. None of them has any competing interest related to this article. The other authors declare that they have no competing interests.

## Authors’ contributions

All authors conceived and designed the study, KL, FB and IA-A contributed to materials and analysis tools. KL, FB and LR analysed the data, KL, FB, LR, IA-A, DLB and TB contributed to data interpretation. All the authors contributed to drafts of the article, commented and contributed to revised drafts of the paper and read and approved the final draft. All authors read and approved the final manuscript.

## Pre-publication history

The pre-publication history for this paper can be accessed here:

http://www.biomedcentral.com/1471-2296/14/85/prepub
